# Artificial intelligence-driven prediction and interpretation of central line-associated bloodstream infections in ICU: insights from the MIMIC-IV database

**DOI:** 10.3389/fpubh.2025.1675077

**Published:** 2025-09-25

**Authors:** Yang He, Jiali Huang, Na Li, Gaosheng Zhou, Jinglan Liu

**Affiliations:** ^1^The First College of Clinical Medical Science, China Three Gorges University, Yichang Central People’s Hospital, Yichang, China; ^2^Intensive Care Unit, Yichang Central People’s Hospital, Yichang, China; ^3^Yichang Clinical Research Center for Sepsis, Yichang, China; ^4^Hubei Provincial Clinical Research Center for Critical Care Medicine, Sepsis Research Collaborative Unit, Yichang, China; ^5^Medical Administration Department, Yichang Central People’s Hospital, Yichang, China; ^6^Department of Nursing, Yichang Central People’s Hospital, Yichang, China

**Keywords:** central line-associated bloodstream infection, ICU, machine learning, risk prediction mode, SHAP

## Abstract

**Objective:**

To develop and internally validate interpretable machine learning (ML) models for predicting individual central line-associated bloodstream infection (CLABSI) risk in adult ICU patients with central venous catheters (CVCs) using the MIMIC-IV database.

**Methods:**

We conducted a retrospective observational cohort study using the MIMIC-IV database. Adult ICU patients with both central venous catheter placement and blood culture evaluation were included. Patients were classified into CLABSI and non-CLABSI cohorts based on central venous catheter tip culture results. A comprehensive set of demographic, physiological, laboratory, therapeutic, and nursing variables was extracted. Feature selection employed Least Absolute Shrinkage and Selection Operator (LASSO) regression. Seven machine learning (ML) models—logistic regression, decision tree, random forest, XGBoost, support vector machine, neural network, and gradient boosting—were developed and compared. Discrimination and calibration were assessed using the area under the receiver operating characteristic curve (AUC), accuracy, sensitivity, specificity, F1 score, and Brier score. The optimal model was interpreted with SHAP (SHapley Additive exPlanations) values to elucidate feature contributions.

**Results:**

Among 11,999 ICU patients, 519 (4.3%) developed CLABSI. CLABSI patients were younger (61.0 vs. 66.0 years), had higher rates of multi-lumen catheters (91.3 vs. 63.6%), mechanical ventilation (90.9 vs. 74.0%), and dialysis (34.9 vs. 7.2%; all *p* < 0.001). The random forest model achieved optimal performance (AUC 0.950, 95% CI 0.931–0.966; sensitivity 0.904, specificity 0.865), outperforming traditional models. SHAP analysis identified ICU length of stay, unique caregivers, and arterial catheterization as top predictors. CLABSI cases exhibited prolonged ICU stays, increased caregiver exposure, and elevated inflammatory markers. Decision curve analysis confirmed clinical utility, with robust performance maintained in sensitivity analyses.

**Conclusion:**

Machine learning models, particularly the random forest model, accurately predict CLABSI risk in ICU patients. The use of interpretable AI techniques such as SHAP enhances transparency and provides actionable insights for clinical practice. These findings support the development of early warning systems to reduce CLABSI incidence and improve patient outcomes.

## 1 Introduction

Central line-associated bloodstream infection (CLABSI) is defined as a laboratory-confirmed bloodstream infection in a patient with a central venous catheter (CVC) in place for more than 48 h prior to the index positive blood culture, with no alternative source identified (1). Despite sustained prevention initiatives, CLABSI remains an enduring challenge in critical care, with reported incidence ranging from 0.8 to 5.7 episodes per 1,000 catheter-days across healthcare systems (2–4). Such variability reflects not only differences in infrastructure and adherence to infection control practices but also limitations in traditional surveillance sensitivity that may underestimate the true burden (2–4). Clinically, CLABSI is associated with a 2.1–3.4-fold increase in mortality and an excess economic burden of approximately US$46,000 per episode, underscoring the need for proactive, individualized risk stratification strategies to guide timely preventive interventions (5).

Conventional risk assessment frameworks—often rule-based or derived from limited multivariable regression models—have limited capacity to represent the dynamic, nonlinear, temporally dependent interactions among multiple factors and are ill-suited for real-time clinical decision support in the intensive care unit (ICU) (6). Machine learning (ML) methods have achieved superior performance over traditional models in predicting complex ICU outcomes—such as sepsis, mortality, and hemodynamic deterioration—by leveraging high-dimensional electronic health record (EHR) data and capturing latent, nonlinear interactions (7–9). Early investigations have applied ML to catheter-related complications (10, 11). However, current CLABSI- or catheter-focused predictive efforts exhibit several methodological and translational gaps: (i) dependence on single-center or narrowly defined cohorts with limited adult ICU specificity (10, 11); (ii) restricted feature spaces with insufficient incorporation of granular nursing-charted, time-varying exposure, and device management variables and (iii) limited explainability, with “black box” outputs impeding clinical trust and actionability (12, 13). These deficiencies hinder integration of predictive tools into daily CLABSI prevention bundles and early warning workflows.

Interpretable AI frameworks—particularly SHapley Additive exPlanations (SHAP)—provide a principled means to decompose model predictions into additive feature contributions, enabling transparent linkage between elevated predicted risk and modifiable care processes (13). Integrating explainability into model development is essential to move from retrospective accuracy benchmarks toward prospectively actionable, clinician-facing decision support.

Therefore, in this study we developed and internally validated a suite of ML models for individualized CLABSI risk prediction in adult ICU patients with CVCs using the MIMIC-IV database. We assembled a comprehensive multidomain feature set encompassing demographic, physiological, laboratory, therapeutic, device-related, and nursing-charted variables, and applied Least Absolute Shrinkage and Selection Operator (LASSO) for feature selection before training seven commonly used algorithms under a standardized evaluation framework. We then applied SHAP analysis to elucidate individual feature contributions, with emphasis on potentially modifiable clinical management factors.

Methodological Aims: We sought to develop and internally validate a suite of interpretable machine learning models for individualized CLABSI risk prediction in adult ICU patients with CVCs using MIMIC-IV, identify the model with optimal discrimination and calibration, and generate transparent feature attributions to inform targeted prevention strategies. We prespecified four hypotheses: (1) ML models would achieve high discrimination (AUC > 0.90) using routinely collected EHR data; (2) a tree-based ensemble (e.g., random forest or gradient boosting) would outperform baseline logistic regression in both discrimination and calibration; (3) SHAP analysis would highlight modifiable or process-related factors (e.g., catheter dwell time, duration of mechanical ventilation prior to CVC placement, arterial catheterization, exposure to specific antibiotic classes, cumulative fluid balance parameters) as major contributors to elevated predicted risk; and (4) the final interpretable model would maintain acceptable calibration (low Brier score) while retaining clinical relevance for integration into early warning workflows.

## 2 Materials and methods

This retrospective observational cohort study used data from the Medical Information Mart for Intensive Care IV (MIMIC-IV) database. MIMIC-IV, developed jointly by the Laboratory for Computational Physiology at the Massachusetts Institute of Technology, Beth Israel Deaconess Medical Center, and Philips Healthcare, is a large, freely accessible, de-identified critical care resource containing comprehensive demographic, laboratory, diagnostic, outcome, and related data on hundreds of thousands of ICU patients admitted between 2008 and 2019 (14). Access to the database was granted after completion of the required online training (author certification ID: 13278787). Because MIMIC-IV is publicly available and fully de-identified, the requirement for institutional review board (IRB) approval was waived.

We included adult ICU patients who had a central venous catheter (CVC) placed and underwent blood culture testing. For patients with multiple ICU stays, only the first admission was analyzed. Exclusion criteria were: (1) age <18 years; (2) missing key demographic information; (3) ICU length of stay <6 h; (4) positive blood cultures collected within 48 h after CVC insertion (to exclude pre-existing bacteremia); and (5) no blood culture data during the observation period; (6) discharge, transfer, or death within 48 h after CVC insertion.

### 2.1 Grouping

Owing to structural limitations of the MIMIC-IV database (e.g., incomplete documentation of catheter removal times and limited clinical context), we operationalized CLABSI classification using simplified blood culture–based criteria aligned with CDC surveillance principles (15). Patients were categorized as CLABSI or non-CLABSI. The CLABSI group comprised patients with at least one pathogenic positive blood culture obtained more than 48 h after central venous catheter (CVC) insertion and before documented CVC removal; when removal time was unavailable, cultures within 30 days of insertion were considered within the at-risk window. Common skin commensals—coagulase-negative staphylococci, Bacillus species (excluding *B. anthracis*), Corynebacterium species, and Cutibacterium (formerly Propionibacterium) species—were classified as true positives only if the same organism was isolated from two or more blood culture sets drawn from separate venipunctures. The non-CLABSI group consisted of patients without qualifying positive blood cultures during the corresponding risk window; single positive isolates of the above commensals (not meeting the duplication criterion) were treated as contaminants and classified as non-CLABSI.

### 2.2 Data extraction and variable collection

We extracted data using Navicat Premium (v16.1.12) and R (v4.3.3), linking tables through the unique stay_id identifier. To ensure temporal integrity and prevent data leakage, all predictor variables were extracted using only data available prior to the outcome determination point. For model development, we established a prediction time point at 48 h after catheter insertion, ensuring all predictor variables were collected using data available up to this time point.

We collected a comprehensive set of patient-level variables. Demographic variables included sex, race, marital status, insurance type, and age. Clinical variables included central venous catheter (CVC) type and insertion site, use of mechanical ventilation, vasopressor administration, arterial catheter placement, renal replacement therapy, antibiotic exposure (antibiotic class and administration within the 48 h preceding catheterization), presence of other concomitant intravascular catheters, and CVC dwell time (for prediction modeling, this was calculated as hours from insertion to the 48-h prediction time point). Additional variables comprised admission weight, body mass index (BMI), hospital length of stay (calculated from admission to the prediction time point), ICU length of stay (calculated from ICU admission to the prediction time point), and duration of mechanical ventilation prior to catheterization (hours).

Vital signs were summarized over a 48 h window (from catheter insertion to 48 h post-insertion) and included temperature (mean, maximum, minimum), heart rate (mean, maximum), systolic and diastolic blood pressure (mean values), mean arterial pressure, respiratory rate (mean), and oxygen saturation (mean, minimum). Laboratory measurements (hematocrit, hemoglobin, platelet count, lactate, creatinine, blood urea nitrogen, sodium, potassium, calcium, prothrombin time [PT], partial thromboplastin time [PTT], international normalized ratio [INR], glucose, and white blood cell count) were abstracted within a −24 to +48 h window relative to catheter insertion.

Fluid intake and output (and net balance, if available), vasopressor dose, nursing charting frequency, number of distinct nursing staff, and nursing-related process indicators (including day vs. night shift insertion) were recorded for the 24 h before catheterization and the first 48 h after catheterization. CLABSI events were ascertained from blood culture results, with the at-risk window defined as >48 h after catheter insertion until catheter removal or day 30, whichever occurred first. All catheter removal times were documented in our dataset.

### 2.3 Statistical analysis

Categorical variables were expressed as n (%), and continuous variables as median (IQR). Group differences were assessed using the Chi-square test (or Fisher’s exact test when expected counts were small) for categorical variables and the Mann–Whitney U test for continuous variables. All statistical tests were two-sided with *p* < 0.05 considered significant. Variables with >20% missing values were excluded. For baseline comparisons, missing values in categorical variables were grouped as “Missing,” while missing values in continuous variables were not imputed.

All remaining candidate variables (without prior univariate *p*-value filtering) entered LASSO logistic regression. Before LASSO, categorical missing values were imputed using the mode and continuous missing values using the median (training set only). Categorical variables were one-hot encoded; continuous variables were standardized. A stratified 5-fold cross-validation procedure selected the optimal lambda for LASSO (alpha = 1). The 15 variables with the largest absolute coefficients (non-zero after regularization) were retained for subsequent modeling.

To address class imbalance, SMOTE (applied only to the training set) was combined with random under-sampling. We compared SMOTE with no balancing (Supplementary Table 1); SMOTE achieved comparable or superior AUC, F1 score, sensitivity, specificity, and Brier score.

**Figure 1:**
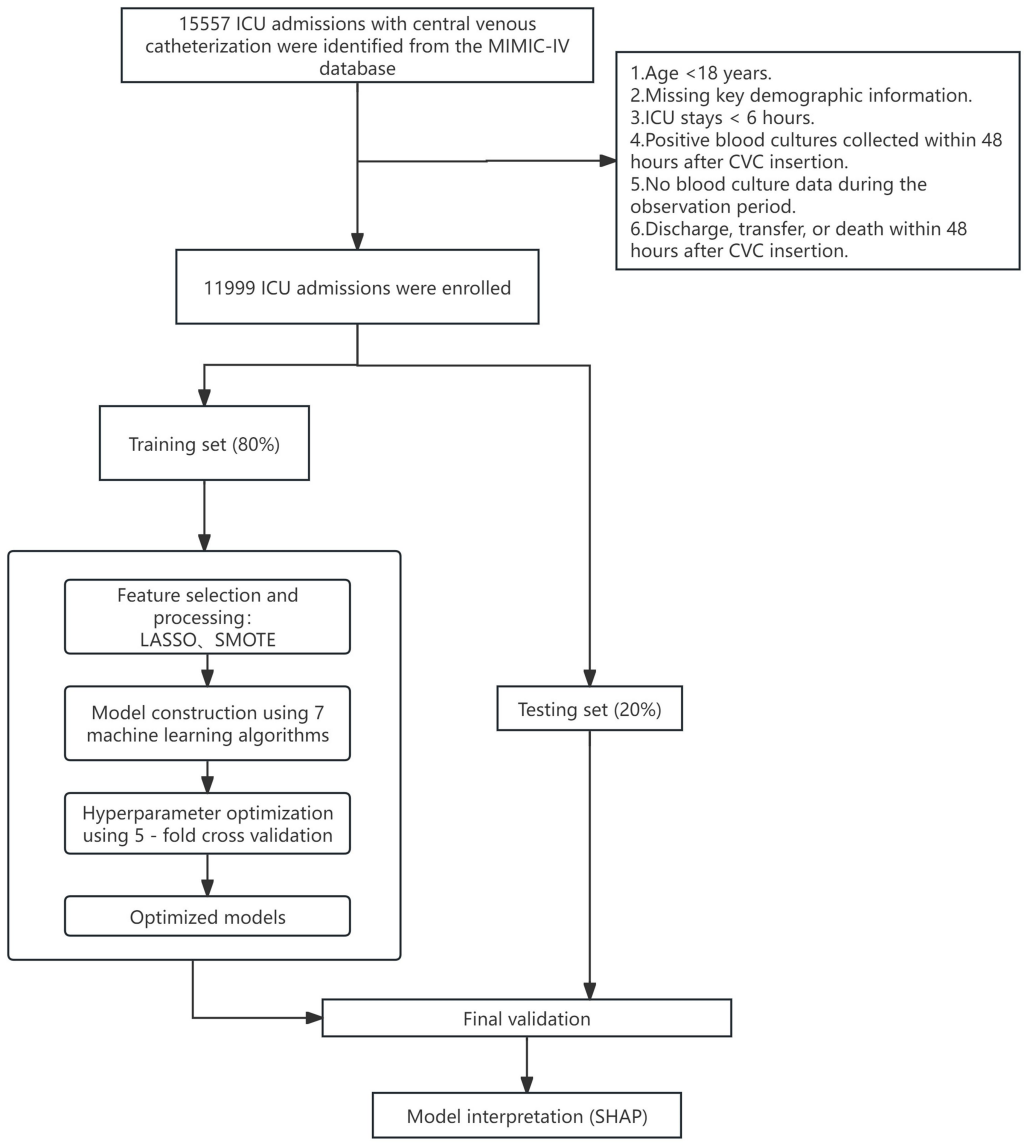
Study flowchart.

Data were split into training and test sets in an 8:2 ratio using stratified random sampling to preserve class distribution. We evaluated seven machine learning algorithms: logistic regression, decision tree, random forest, XGBoost, support vector machine (SVM), artificial neural network (ANN), and gradient boosting machine (GBM). To ensure robust model selection and unbiased performance estimation, we implemented nested cross-validation, where the outer loop (5-fold) assessed generalization performance and the inner loop (3-fold) optimized hyperparameters. For models with extensive parameter spaces (>50 combinations), we employed randomized search with 50 iterations; otherwise, we conducted exhaustive grid search. Hyperparameter grids were tailored to each algorithm and included regularization parameters (C for logistic regression and SVM; alpha for neural networks), tree-based parameters (max_depth, n_estimators, min_samples_split, min_samples_leaf, and learning_rate for gradient boosting methods), SVM-specific parameters (kernel type, gamma for RBF kernel), neural network architecture (hidden_layer_sizes, activation functions, learning_rate_init), and class imbalance handling strategies (class_weight for applicable models, scale_pos_weight for XGBoost). Each algorithm’s final model was retrained on the entire training set using the hyperparameter configuration most frequently selected across outer folds. Model performance was comprehensively evaluated using area under the receiver operating characteristic curve (AUC), accuracy, sensitivity, specificity, F1 score (with decision threshold optimized on the training set), and Brier score for calibration assessment. We calculated 95% confidence intervals through 1,000 bootstrap resamples of the test set. Model calibration and clinical utility were further assessed via calibration plots and decision curve analysis. The best-performing model was interpreted using SHapley Additive exPlanations (SHAP), applying TreeExplainer for tree-based models and KernelExplainer for others. All analyses were implemented in Python (v3.10.0) using scikit-learn, XGBoost, and SHAP libraries. The complete analytical workflow is illustrated in Figure 1.

## 3 Results

### 3.1 Baseline characteristics

Among 11,999 ICU patients undergoing central venous catheterization, 519 (4.3%) developed CLABSI. Compared with those without CLABSI, affected patients were younger (median age 61.0 vs. 66.0 years, *p* < 0.001); more frequently identified as non-White (42.2 vs. 32.3%, *p* < 0.001); more often married (60.3 vs. 51.2%, *p* < 0.001); and more frequently covered by Medicaid or other insurance types (23.5 vs. 16.9%, *p* < 0.001). They also had greater use of multi-lumen catheters (91.3 vs. 63.6%) and higher frequencies of left internal jugular (22.5 vs. 7.9%), left subclavian (13.9 vs. 5.8%), and right subclavian (12.1 vs. 4.5%) venous access (all *p* < 0.001). Rates of mechanical ventilation (90.9 vs. 74.0%), vasopressor use (67.4 vs. 49.8%), arterial catheterization (80.3 vs. 63.7%), and dialysis (34.9 vs. 7.2%) were likewise higher (all *p* < 0.001). Pre-catheter antibiotic exposure within 48 h was more common (83.2 vs. 58.2%, *p* < 0.001), with use of a broader range of antibiotic classes. Catheter dwell time (median 48.00 vs. 46.65 h), hospital length of stay (29.73 vs. 9.95 days), and ICU length of stay (20.81 vs. 3.32 days) were markedly longer (all *p* < 0.001). The CLABSI group further demonstrated higher temperature, heart rate, platelet count, lactate, renal function parameters, coagulation parameters, glucose, and white blood cell count (all *p* < 0.001). Peri-catheter 24-h fluid input and output (pre- and post-catheterization windows) were greater, and the number of distinct caregivers was higher (median 36 vs. 10, p < 0.001). Detailed baseline characteristics are provided in Supplementary Table 2.

### 3.2 Selected predictive features

**Figure 2:**
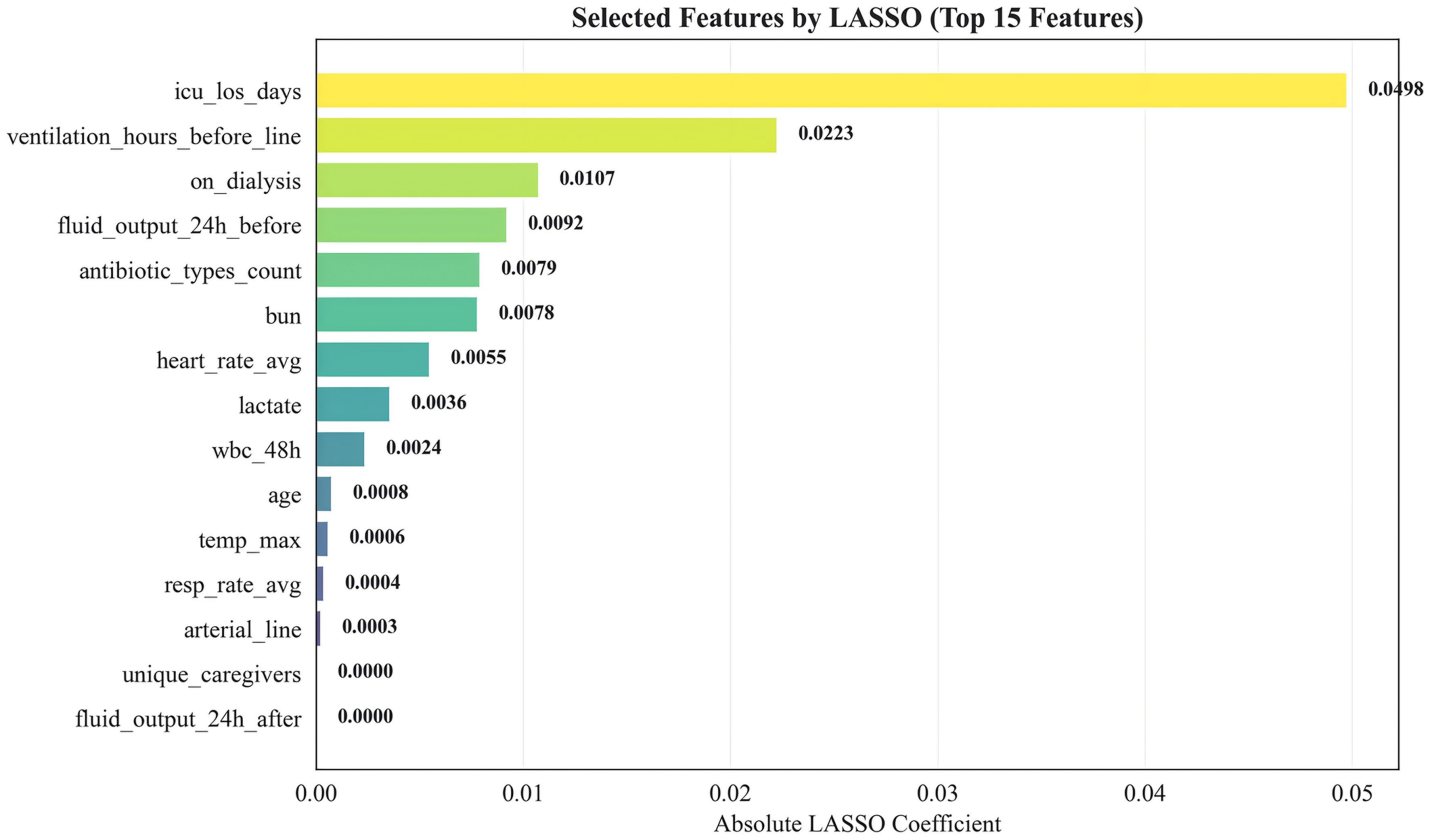
Feature selection using LASSO.

We applied LASSO logistic regression for feature selection. Figure 2 presents the top 15 selected features ranked by the absolute magnitude of their LASSO coefficients. ICU length of stay (icu_los_days) contributed the largest absolute coefficient, followed by pre-catheter mechanical ventilation duration (ventilation_hours_before_line) and dialysis status (on_dialysis). Other influential features included 24-h pre-catheter fluid output, number of antibiotic classes used, blood urea nitrogen (BUN), mean heart rate, lactate, white blood cell count within the preceding 48 h, age, maximum body temperature, mean respiratory rate, arterial catheterization, unique caregivers, and 24-h post-catheter fluid output. This feature set was subsequently used for model construction.

### 3.3 Model performance

We evaluated multiple machine learning algorithms for CLABSI prediction (Table 1). Tree-based ensemble models consistently outperformed single learners, with the random forest achieving the strongest overall performance. The random forest attained an AUC of 0.950 (95% CI 0.931–0.966), sensitivity 0.904 (95% CI 0.863–0.943), specificity 0.865 (95% CI 0.823–0.900), F1 score 0.858, and Brier score 0.088. Its PLR was 6.693 (95% CI 5.069–9.359) and NLR 0.111 (95% CI 0.065–0.158); PPV and NPV were 0.817 and 0.931, respectively—collectively indicating excellent discrimination and good calibration. Calibration analysis showed lower Brier scores for the random forest, XGBoost, and gradient boosting (0.088, 0.093, and 0.098) than for traditional models, confirming closer alignment between predicted risks and observed outcomes. Decision curve analysis further demonstrated that these ensemble models yielded the highest net benefit across most clinically relevant thresholds, substantially exceeding logistic regression and the single decision tree and supporting superior potential clinical utility.

**Table 1:** Performance comparison of different machine learning models in predicting.

Models	AUC (95%CI)	Cutoff value	SEN (95%CI)	SPE (95%CI)	PLR (95%CI)	NLR (95%CI)	PPV (95%CI)	NPV (95%CI)	F1 score	Brier score
Logistic Regression	0.910 (0.888–0.932)	0.258	0.760 (0.704–0.812)	0.868 (0.833–0.900)	5.762 (4.395–7.485)	0.277 (0.218–0.342)	0.794 (0.741–0.845)	0.844 (0.811–0.885)	0.776	0.124
Decision Tree	0.831 (0.796–0.868)	0.429	0.822 (0.774–0.872)	0.833 (0.789–0.870)	4.917 (3.890–6.466)	0.214 (0.154–0.277)	0.767 (0.713–0.821)	0.875 (0.840–0.912)	0.794	0.160
Random Forest	0.950 (0.931–0.966)	0.480	0.904 (0.863–0.943)	0.865 (0.823–0.900)	6.693 (5.069–9.359)	0.111 (0.065–0.158)	0.817 (0.768–0.874)	0.931 (0.902–0.959)	0.858	0.088
XGBoost	0.949 (0.933–0.963)	0.455	0.885 (0.847–0.924)	0.875 (0.837–0.909)	7.054 (5.484–9.879)	0.132 (0.086–0.178)	0.825 (0.764–0.874)	0.919 (0.890–0.950)	0.854	0.093
SVM	0.919 (0.897–0.940)	0.446	0.856 (0.797–0.904)	0.830 (0.795–0.872)	5.022 (4.017–6.601)	0.174 (0.116–0.242)	0.771 (0.717–0.823)	0.896 (0.857–0.928)	0.811	0.113
Artificial Neural Network	0.922 (0.897–0.943)	0.070	0.889 (0.846–0.929)	0.849 (0.810–0.887)	5.885 (4.680–7.936)	0.130 (0.084–0.185)	0.797 (0.750–0.850)	0.920 (0.889–0.952)	0.841	0.106
Gradient Boosting	0.937 (0.916–0.955)	0.349	0.856 (0.799–0.895)	0.852 (0.809–0.888)	5.786 (4.481–7.556)	0.169 (0.124–0.235)	0.795 (0.737–0.847)	0.898 (0.858–0.928)	0.824	0.098

**Figure 3:**
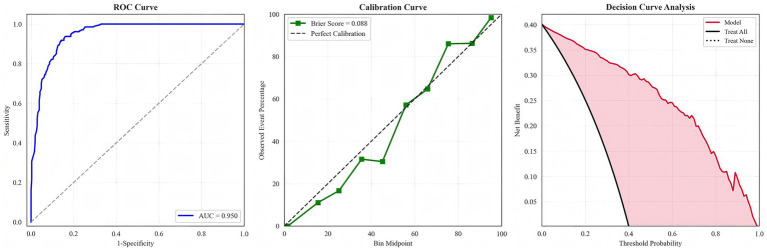
Random forest evaluation.

Because arterial catheters can themselves serve as a source of bloodstream infection, raising the possibility that some arterial catheter–related events were misattributed to CLABSI, we conducted a prespecified sensitivity analysis excluding patients with arterial catheterization. Performance metrics for all models, including the random forest, remained materially unchanged (Supplementary Table 3), underscoring the robustness of the primary findings. To emphasize the principal results, only the ROC, calibration, and decision curves of the best-performing random forest model are presented in the main text (Figure 3); curves for the remaining models are provided in Supplementary Figures 1–6.

### 3.4 SHAP analysis for feature importance

**Figure 4:**
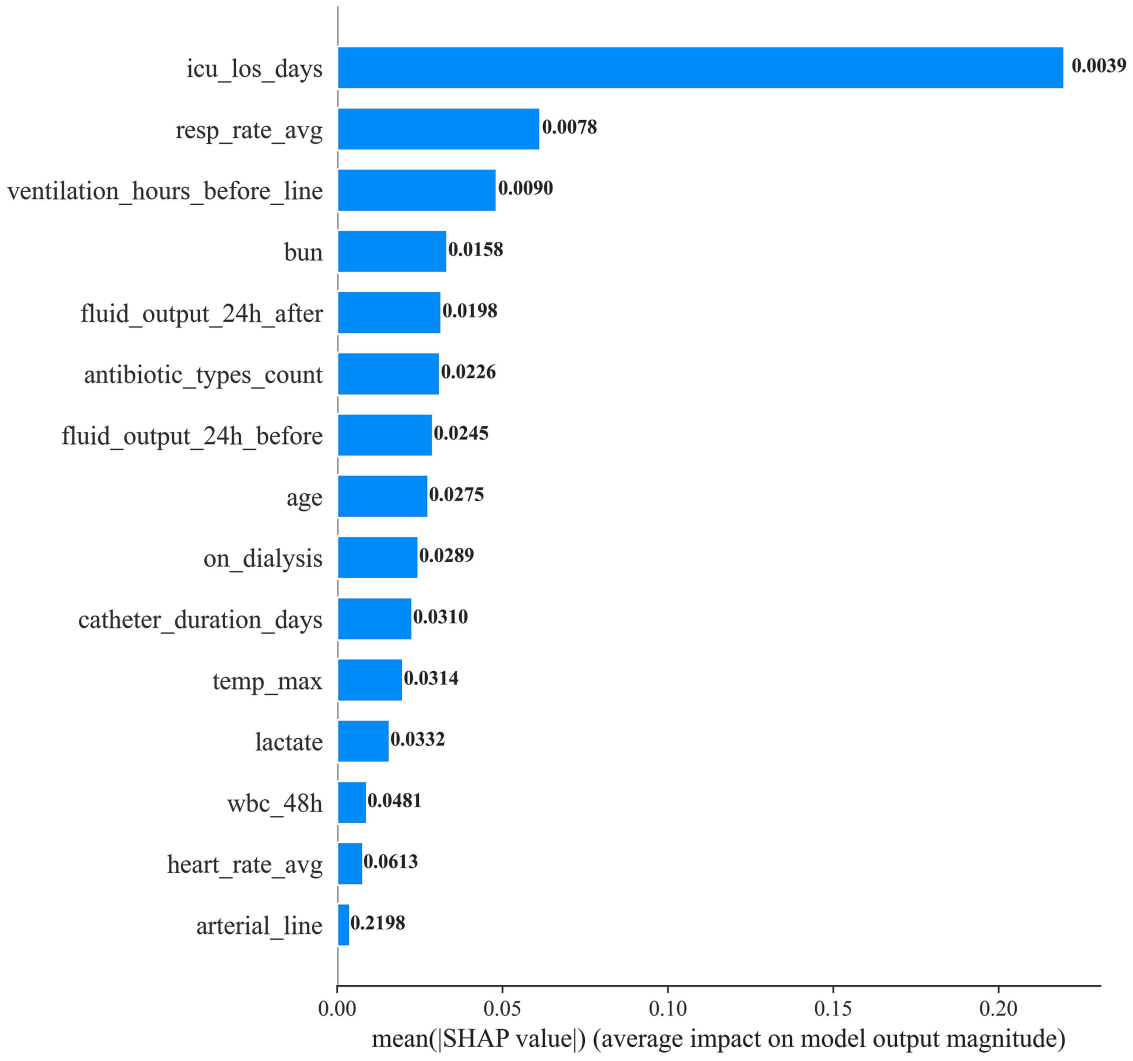
SHAP feature importance bar plot.

**Figure 5:**
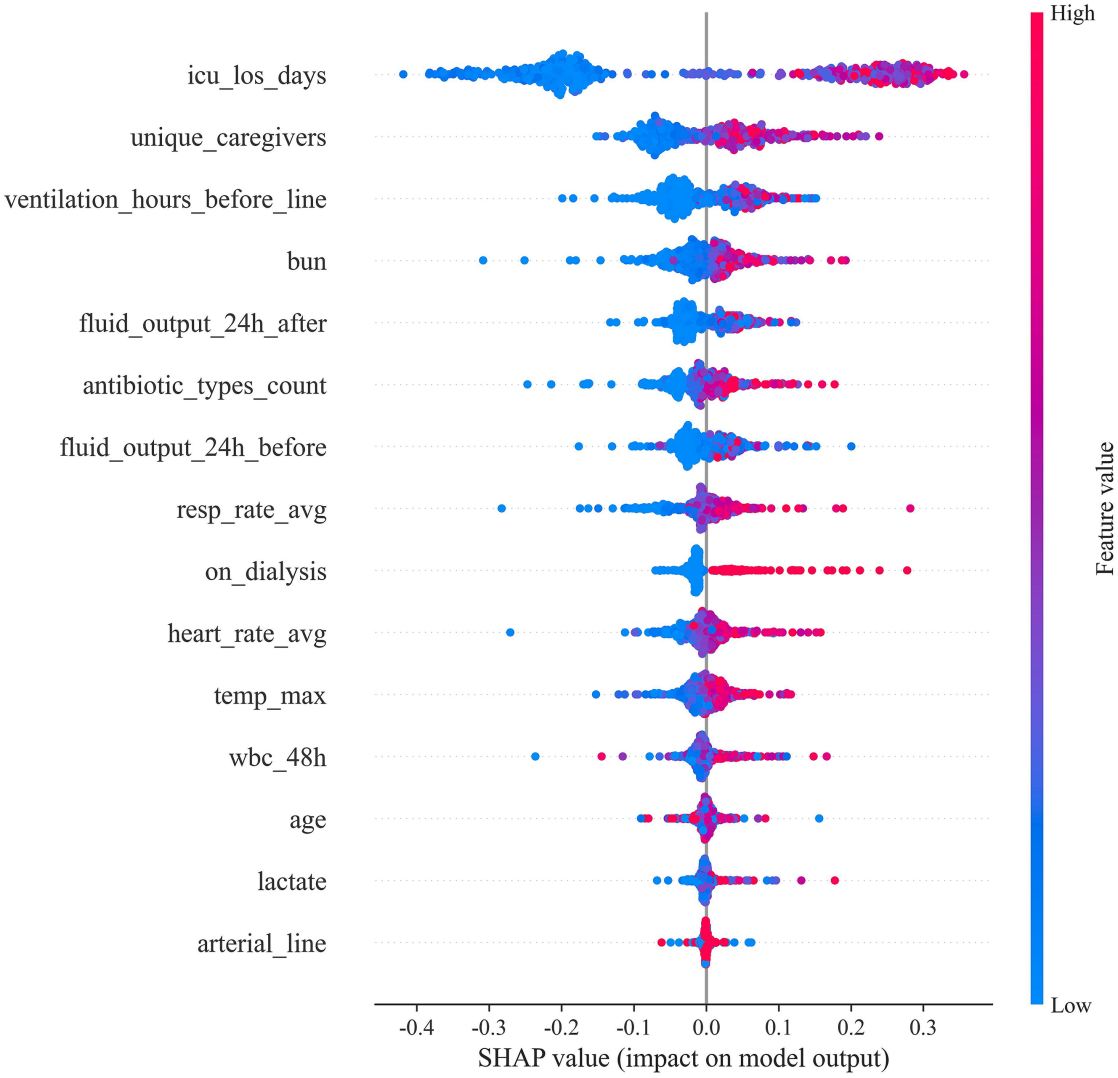
SHAP Beeswarm plot.

To elucidate how individual predictors drive CLABSI risk estimates, we applied SHAP to the final random forest model (Figures 4, 5). ICU length of stay (icu_los_days) exerted the greatest influence on predicted risk, followed by the number of unique caregivers (unique_caregivers), arterial catheterization (arterial_line), heart rate average (heart_rate_avg), maximum temperature (temp_max), and white blood cell count within the specified 48-h window (wbc_48h). Other impactful features included blood urea nitrogen (BUN), lactate, age, fluid output (both before and after 24 h of catheter insertion), whether the patient was on dialysis (on_dialysis), average respiratory rate (resp_rate_avg), duration of mechanical ventilation before catheter insertion (ventilation_hours_before_line), and the number of distinct antibiotic classes administered (antibiotic_types_count).

Figure 5 provides the ranked importance profile and corresponding feature effect (dependence) plots. Prolonged ICU stay, a higher number of caregivers, and the presence of an arterial line exhibited monotonic positive associations with predicted CLABSI risk (clustered red points at higher SHAP values). In contrast, fluid output demonstrated non-linear, bidirectional influences, suggesting threshold or interaction effects. Higher heart rate, temperature, and white blood cell counts similarly shifted predictions toward higher risk, while other features showed varying degrees of impact.

### 3.5 Individual-level SHAP force plot interpretation

**Figure 6:**
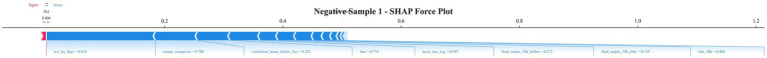
SHAP force plot negative sample 1.

**Figure 7:**
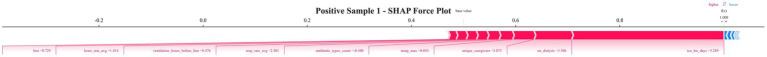
SHAP force plot positive sample 1.

To further illustrate the model’s individual-level decision process, we randomly selected several CLABSI-positive and CLABSI-negative cases and visualized them using SHAP force plots (Figures 6, 7, with additional examples in Supplementary Figures 7–14). In negative cases, shorter ICU length of stay, fewer unique caregivers, shorter duration of mechanical ventilation before catheter insertion, lower bun, lower average heart rate, lower fluid output within 24 h before and after catheterization, and lower wbc within 48 h (blue, pushing the prediction toward lower risk) were the main factors contributing to a negative classification. Physiological and laboratory parameters, including these variables, further decreased the predicted risk. Overall, the net contribution of SHAP values was predominantly negative. Conversely, in positive cases, prolonged ICU length of stay, a greater number of unique caregivers, presence of dialysis, increased average respiratory rate, longer mechanical ventilation prior to catheterization, higher average heart rate, bun, higher maximum temperature, and a greater number of antibiotic types (red, pushing the prediction toward higher risk) were the primary drivers of higher predicted CLABSI probability. In some cases, these variables provided additional positive SHAP values, further increasing the risk assessment.

## 4 Discussion

### 4.1 Main findings and model performance

In this study, we developed and validated a machine-learning–based model to predict CLABSI risk using data from 11,999 ICU patients, among whom 519 (4.3%) developed CLABSI. The random forest model demonstrated excellent discrimination (AUC 0.950; 95% CI 0.931–0.966), outperforming logistic regression (AUC 0.910). These findings highlight the potential—rather than guaranteed—advantages of ensemble methods for high-dimensional clinical prediction. Consistent with Fu et al. (11), our results reinforce the capacity of ensemble approaches to capture nonlinear relationships and higher-order interactions.

Recent evaluations of electronic health record modeling frameworks have compared binary, survival, multinomial, and competing-risk random forests for predicting hospital-acquired infections, including CLABSI (16). While survival and competing-risk models can more fully represent event timing and competing outcomes, binary classifiers remain predominant in practice due to parsimony and computational efficiency, with little loss in discrimination. Our results concur, showing that a binary random forest yields strong CLABSI risk discrimination while preserving implementation efficiency.

The CLABSI incidence was 4.3%, consistent with published rates (0.8–5.7 per 1,000 catheter-days) (2, 3). Patients with CLABSI exhibited markedly longer catheter dwell time (median 48.00 vs. 46.65 h), and prolonged hospital (29.73 vs. 9.95 days) and ICU stays (20.81 vs. 3.32 days). These associations likely reflect both underlying severity and the impact of CLABSI itself. Given the observational design, temporal directionality and causality cannot be inferred.

### 4.2 Model interpretability and risk factor insights

SHAP analysis delineated a hierarchy of risk determinants, with ICU length of stay (LOS) the dominant predictor. ICU LOS likely embodies both severity-of-illness burden and cumulative exposure to nosocomial hazards—invasive procedures, pathogen load, and evolving immune dysfunction (17–19). Prior work similarly demonstrates nonlinear escalation of adverse outcome risk beyond approximately seven ICU days, though residual confounding and time-dependent bias may partially shape this trajectory (20, 21).

Pre-catheterization mechanical ventilation duration showed a strong, non-causal association with CLABSI risk. Rather than implying that ventilation causes CLABSI, it operates as a proxy for severity and invasive care intensity. Sedation-related immobility, repeated airway manipulation, and ventilator-associated pneumonia may jointly amplify systemic inflammation and compromise catheter site defenses (18, 22). Unmeasured confounders likely contribute and justify prospective mechanistic evaluation (23, 24).

Fluid balance added incremental stratification value. Fluid overload (>10% positive balance) has been linked to glycocalyx disruption and endothelial injury, plausibly facilitating catheter colonization (25, 26). Capillary leak–related third spacing may further hinder immune cell trafficking and antibiotic penetration at insertion sites (27). These data generate a testable hypothesis that conservative or optimized fluid strategies could mitigate risk, pending interventional confirmation.

Greater pre-catheterization antibiotic exposure was positively associated with CLABSI but should not be interpreted as causal. Broad-spectrum or prolonged regimens may select for resistant flora and induce dysbiosis (22, 28). The median of three antibiotic classes in CLABSI cases versus one in controls likely marks clinical complexity or underlying susceptibility rather than a direct pathogenic effect.

Model-derived interaction patterns underscore compounded risk in multi-system dysfunction: concurrent dialysis (34.9%) and ventilation suggested an approximately multiplicative contribution within the model’s predictive structure, without implying biological synergy. Elevated use of multi-lumen catheters (91.3 vs. 63.6%) and subclavian access similarly aligned with higher predicted risk, potentially reflecting device complexity, indication bias, operator factors, or residual confounding.

### 4.3 Innovation

A principal innovation of this work is embedding SHAP-based interpretability directly within the model development pipeline. SHAP force plots generate patient-level decomposition of predicted risk, furnishing clinicians with a transparent account of how specific features elevate or suppress estimated CLABSI probability and thereby alleviating black-box concerns. The high negative predictive value (93.1% at the observed prevalence) supports a rule-out application that could curtail unwarranted catheter removals, while EHR integration offers a pathway to real-time, continuously refreshed risk stratification. Decision curve analysis demonstrated net clinical benefit across threshold probabilities of 10–60%; definitive impact on practice, however, awaits prospective implementation studies. SHAP-informed rankings are explicitly hypothesis-generating and may guide tailored prevention measures—for example, intensified surveillance or bundle reinforcement in patients with mechanical ventilation >72 h and positive fluid balance >2 L/24 h. Exploratory inclusion of nursing process-of-care indicators (e.g., catheter check frequency, number of providers) operationalizes care quality and suggests opportunities to optimize catheter maintenance protocols and early antifungal prophylaxis. These indicators contributed incremental predictive signal while yielding implementation-oriented, hypothesis-generating insights for nursing practice improvement.

### 4.4 Limitations

SHAP does not confer causal identification. Its values approximate marginal feature contributions under model- and data-dependent distributional assumptions and can become variance-prone with strong collinearity, out-of-distribution feature combinations, or compression of dynamic trajectories into static aggregates (29). Interaction structure may be understated unless explicit interaction attributions are calculated, and pronounced non-linear patterns risk overinterpretation as mechanistic pathways. Independence assumptions in certain computational backends can distort attribution when features are correlated. Consequently, SHAP outputs should be triangulated with clinical reasoning, perturbation/sensitivity analyses, and formal causal inference methods rather than treated as definitive etiologic evidence. A critical limitation concerns the temporal definition of CLABSI and its implications for model applicability and interpretability. By adhering to the CDC definition requiring ≥48 h of catheterization for CLABSI diagnosis, our cohort construction inherently introduces classification bias: patients with catheter dwell times less than 48 h are systematically classified as non-CLABSI cases, regardless of their true infection risk trajectory. This design constraint means our model lacks positive CLABSI examples for early catheter removal scenarios, potentially biasing risk estimates downward for patients whose catheters are removed before the 48-h threshold. Consequently, the model cannot reliably assess infection risk for patients with anticipated short-duration catheterizations, limiting its utility in clinical scenarios where early catheter removal is planned or indicated. In real-world clinical practice, this temporal constraint has important implications for model deployment and interpretation. The risk predictions are most valid for patients expected to have prolonged catheterization (>48 h), while assessments for patients with anticipated shorter catheter durations should be interpreted with caution. Furthermore, computing CLABSI risk after catheter removal may have limited clinical utility, as preventive interventions are no longer actionable post-removal. Important contextual determinants—catheter maintenance quality, hand hygiene adherence, and environmental or workflow factors—were unavailable in the MIMIC database. Class imbalance (4.3% CLABSI) persists as a potential source of performance drift despite SMOTE rebalancing. Omission of microbiological determinants (e.g., biofilm phenotypes) and granular procedural/nursing metrics (e.g., insertion attempt count) constrains mechanistic resolution. Residual confounding, indication bias, and potential immortal time bias tied to time-dependent exposures (e.g., pre-catheter ventilation duration) may influence observed associations.

### 4.5 Future directions

(1) rigorous external validation across heterogeneous ICUs and healthcare systems; (2) prospective evaluation of decision support integration and its effect on CLABSI incidence, antimicrobial use, and patient-centered outcomes; (3) adoption of temporal deep learning architectures (e.g., transformer or sequence models) and potential fusion with imaging or waveform data; (4) application of causal inference (target trial emulation, marginal structural models) to disentangle exposure–outcome relationships; (5) incorporation of smart catheter sensor streams plus high-resolution nursing documentation, tested via stepped-wedge or hybrid effectiveness–implementation designs; (6) health economic modeling to define cost-effectiveness and budget impact; and (7) prospective analyses with time-updated covariates and competing risk frameworks to minimize immortal time and informative censoring biases while refining temporal attribution.

## 5 Conclusion

We built a random forest–based model for CLABSI risk that demonstrated strong predictive performance and, through SHAP, delivered individualized risk attribution. Its integration into clinical workflows may support earlier targeted prevention and more efficient resource use. Prospective external studies are required to establish real-world generalizability, calibration, and clinical impact.

## Data Availability

Publicly available datasets were analyzed in this study. This data can be found at: https://physionet.org/content/mimiciv/2.2/.
