# Supplementary material for: Artificial intelligence-driven prediction and interpretation of central line-associated bloodstream infections in ICU: insights from the MIMIC-IV database
Source: Front Public Health. 2025 Sep 25;13:1675077. doi: 10.3389/fpubh.2025.1675077 (PMC12507818; doi:10.3389/fpubh.2025.1675077)
Supplement: Supplementary file 1 [file Supplementary_file_1.docx]

Supplementary Material

# Supplementary Figures and Tables

## Supplementary Figures


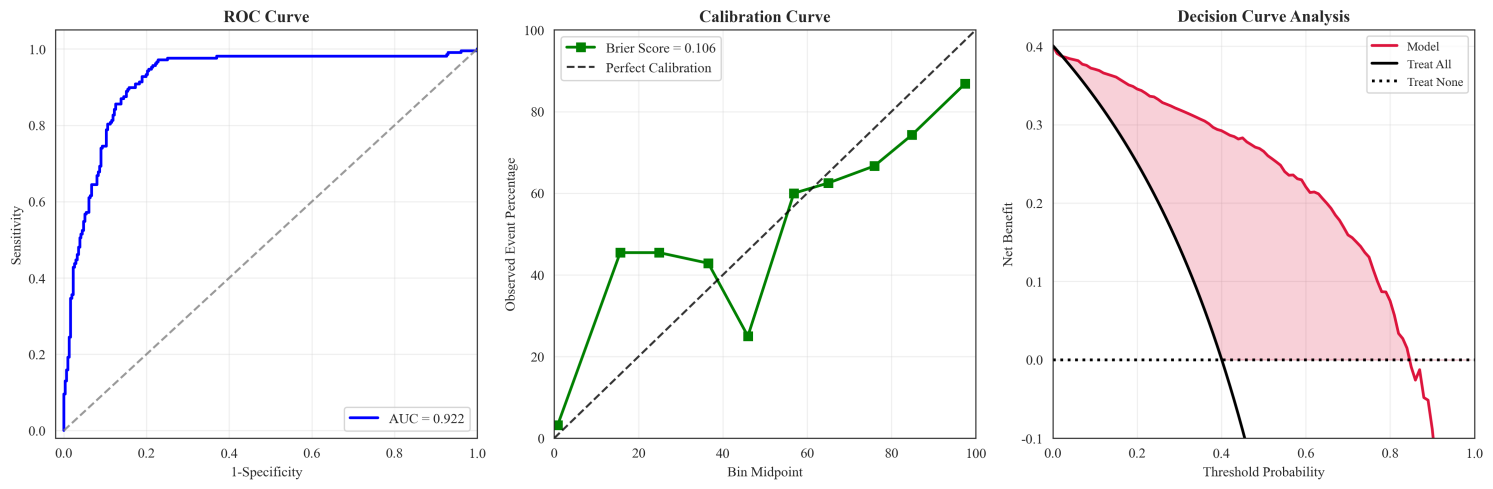


## **Supplementary Figure 1. Artificial Neural Network evaluation.**


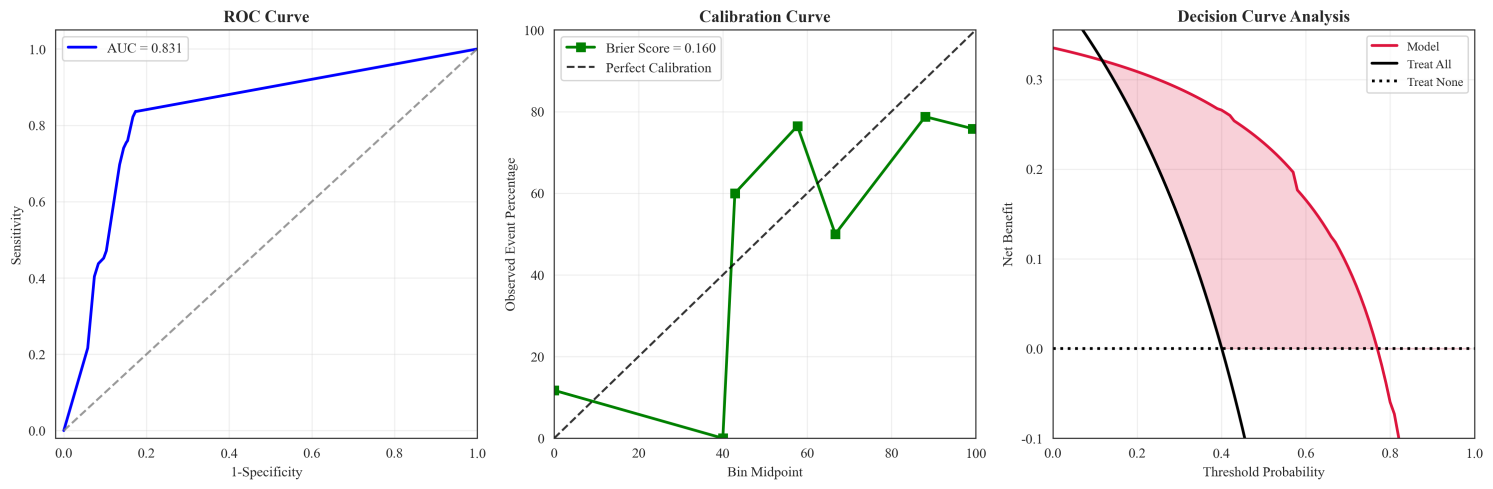


## **Supplementary Figure 2. Decision Tree evaluation.**

##
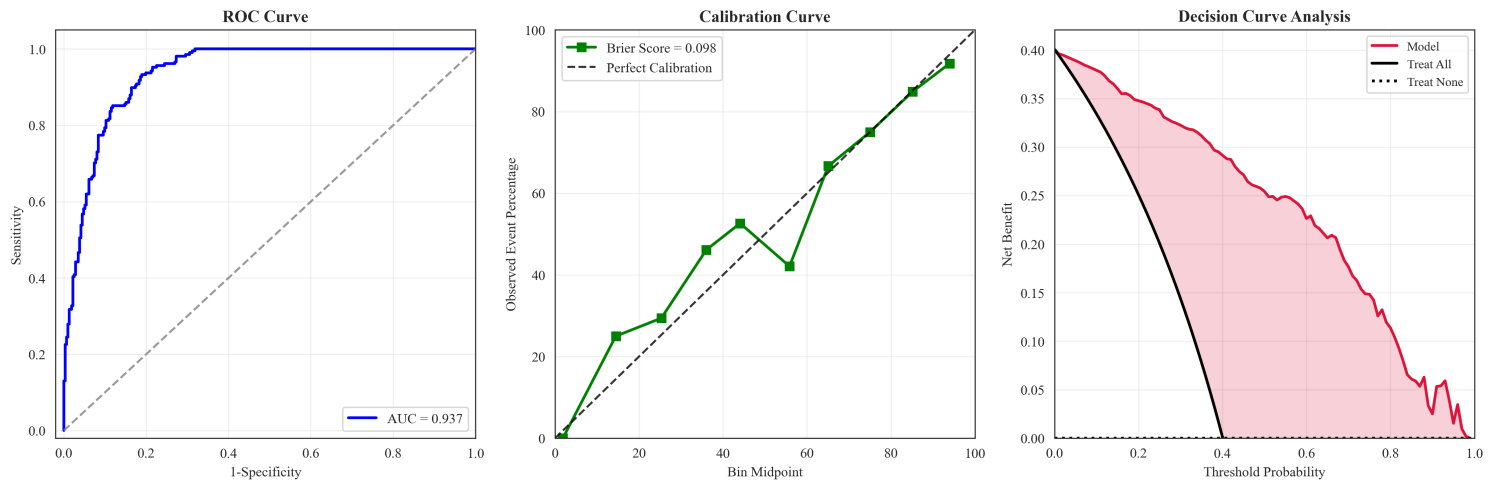


**Supplementary Figure 3. Gradient Boosting evaluation.**


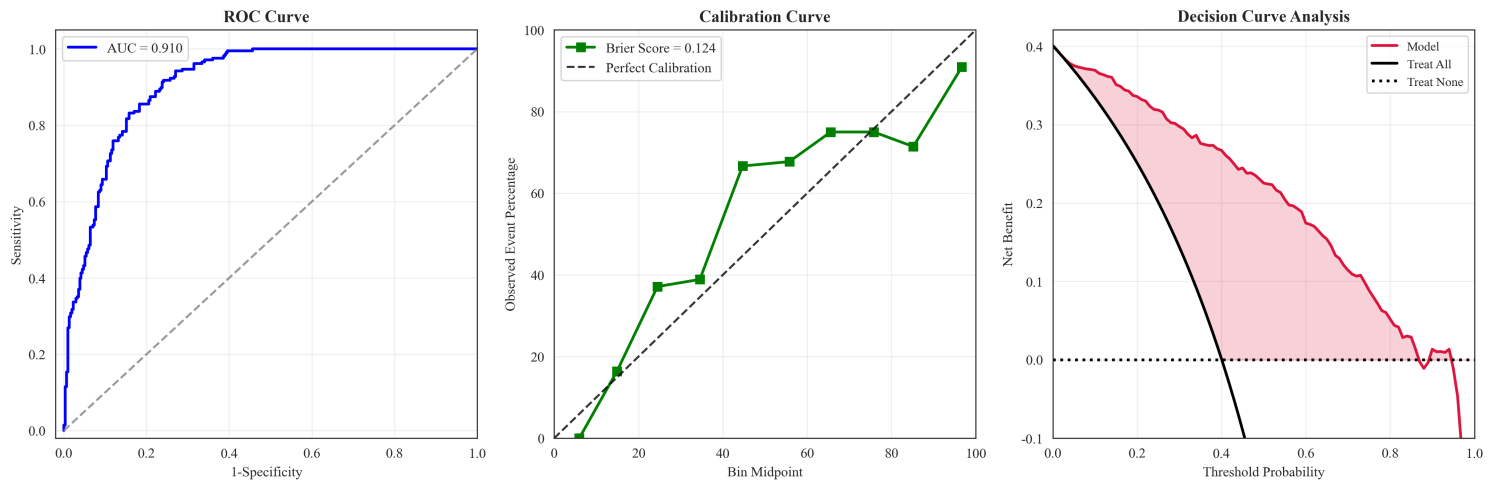


**Supplementary Figure 4. Logistic Regression evaluation.**


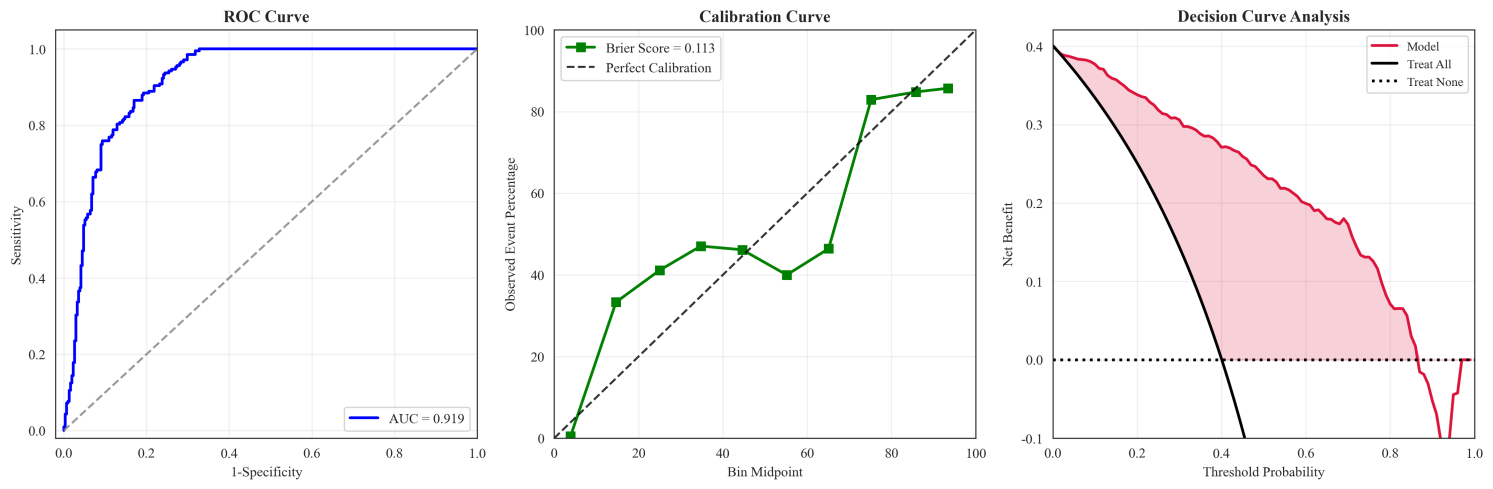


**Supplementary Figure 5. SVM evaluation.**


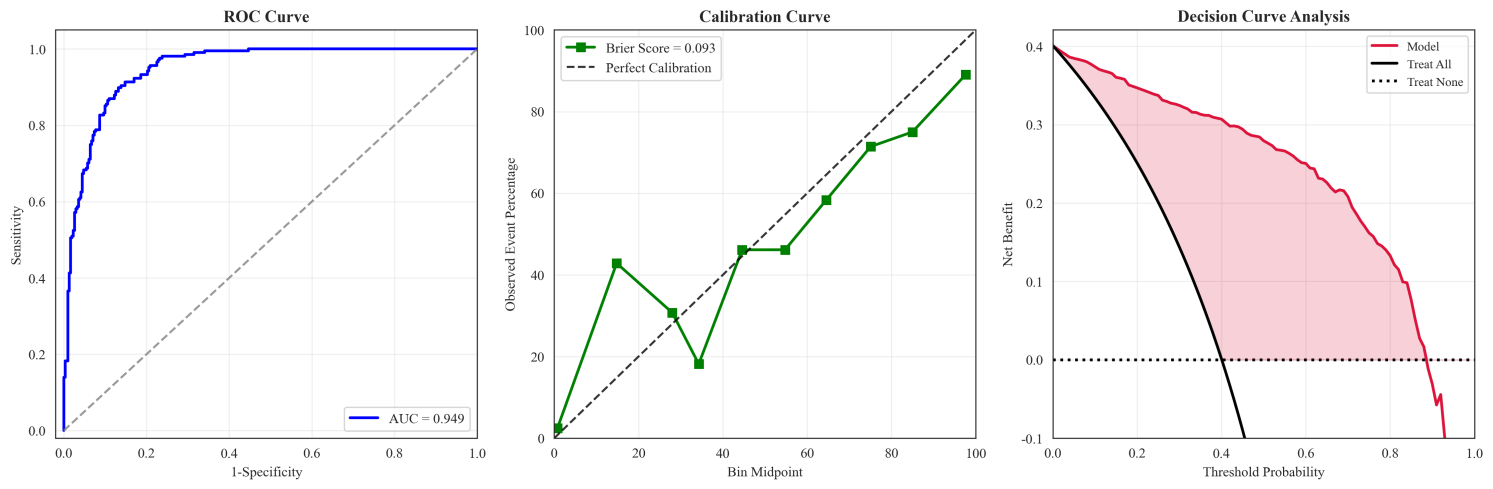


**Supplementary Figure 6. XGBoost evaluation.**


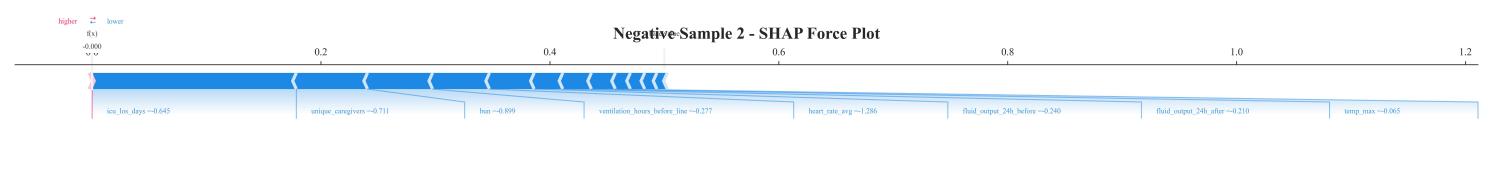


**Supplementary Figure 7. SHAP Force Plot negative sample 2.**


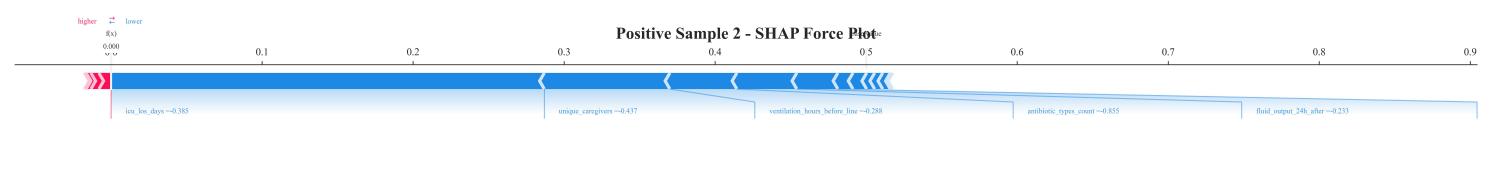


**Supplementary Figure 8. SHAP Force Plot positive sample 2.**


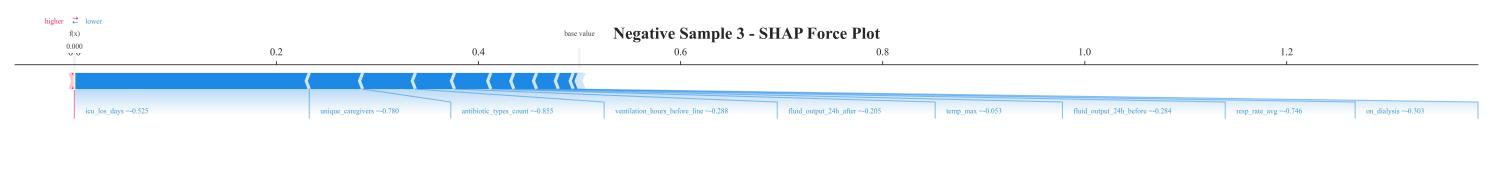


**Supplementary Figure 9. SHAP Force Plot negative sample 3.**


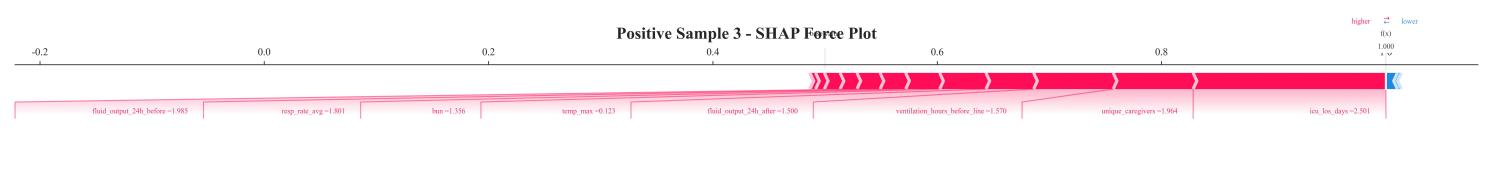


**Supplementary Figure 10. SHAP Force Plot positive sample 3.**


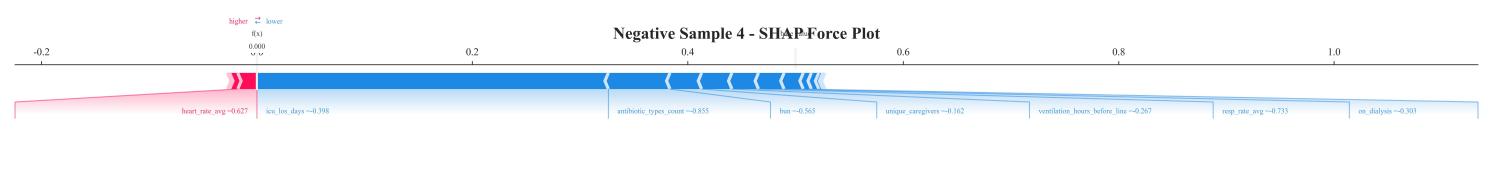


**Supplementary Figure 11. SHAP Force Plot negative sample 4.**


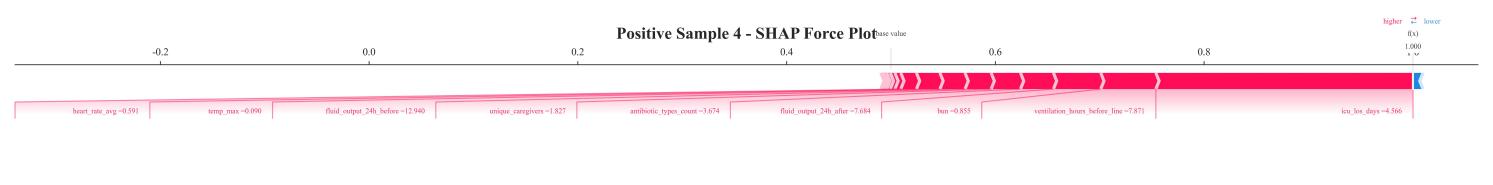


**Supplementary Figure 12. SHAP Force Plot positive sample 4.**


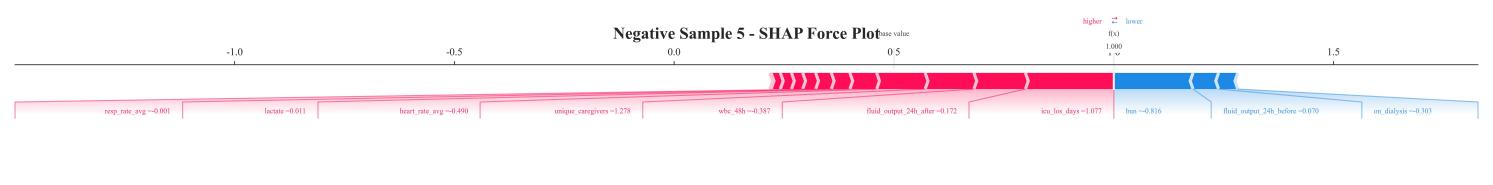


**Supplementary Figure 13. SHAP Force Plot negative sample 5.**


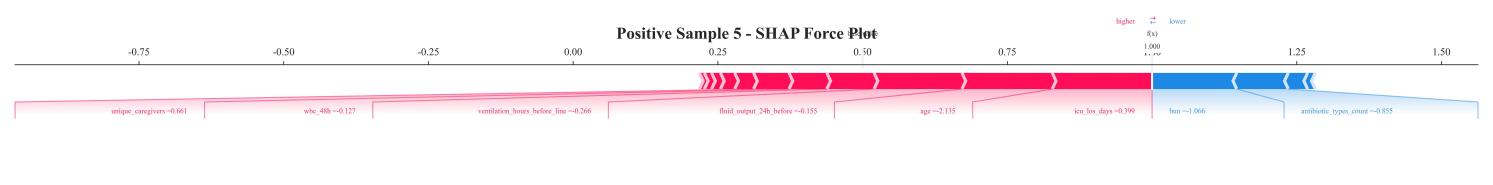


**Supplementary Figure 14. SHAP Force Plot positive sample .**

## Supplementary Tables

| **Models** | **AUC (95%CI)** | **Cutoff value** | **SEN (95%CI)** | **SPE (95%CI)** | **PLR (95%CI)** | **NLR (95%CI)** | **PPV (95%CI)** | **NPV (95%CI)** | **F1 score** | **Brier score** |
| --- | --- | --- | --- | --- | --- | --- | --- | --- | --- | --- |
| Logistic Regression | 0.900 (0.870–0.919) | 0.030 | 0.154 (0.096–0.221) | 0.993 (0.990–0.997) | 22.077 (11.334–52.048) | 0.852 (0.785–0.910) | 0.500 (0.322–0.710) | 0.963 (0.954–0.969) | 0.235 | 0.034 |
| Decision Tree | 0.782 (0.729–0.828) | 0.021 | 0.212 (0.126–0.296) | 0.981 (0.975–0.986) | 11.038 (6.438–17.697) | 0.804 (0.716–0.891) | 0.333 (0.207–0.450) | 0.965 (0.957–0.973) | 0.259 | 0.048 |
| Random Forest | 0.918 (0.896–0.946) | 0.050 | 0.067 (0.019–0.119) | 0.997 (0.994–0.999) | 22.077 (6.105–66.575) | 0.936 (0.885–0.984) | 0.500 (0.214–0.750) | 0.959 (0.951–0.967) | 0.119 | 0.033 |
| XGBoost | 0.911 (0.888–0.932) | 0.002 | 0.135 (0.081–0.200) | 0.994 (0.991–0.997) | 23.775 (11.427–48.352) | 0.870 (0.806–0.926) | 0.519 (0.333–0.706) | 0.962 (0.955–0.968) | 0.214 | 0.036 |
| SVM | 0.773 (0.720–0.842) | 0.039 | 0.019 (0.000–0.044) | 1.000 (1.000–1.000) | 999.999 | 0.981 (0.956–1.000) | 1.000 (0.000–1.000) | 0.957 (0.949–0.966) | 0.038 | 0.037 |
| Artificial Neural Network | 0.888 (0.868–0.911) | 0.005 | 0.192 (0.120–0.261) | 0.983 (0.979–0.989) | 11.619 (7.456–19.498) | 0.821 (0.751–0.892) | 0.345 (0.239–0.471) | 0.964 (0.956–0.972) | 0.247 | 0.042 |
| Gradient Boosting | 0.926 (0.908–0.945) | 0.026 | 0.106 (0.051–0.170) | 0.995 (0.993–0.997) | 22.077 (9.126–51.467) | 0.899 (0.833–0.954) | 0.500 (0.278–0.696) | 0.961 (0.953–0.969) | 0.175 | 0.034 |

**Supplementary Table 1. Model Performance on the Original Imbalanced Dataset (No Class Balancing)**

| Characteristic | Category | Total (n=11999) | CLABSI (n=519, 4.3%) | Non-CLABSI (n=11480, 95.7%) | P-value |
| --- | --- | --- | --- | --- | --- |
| Line Type, n (%) | RIC | 2 (0.0) | 0 (0.0) | 2 (0.0) | <0.001 |
|  | PICC | 10 (0.1) | 0 (0.0) | 10 (0.1) |  |
|  | Hickman | 30 (0.3) | 4 (0.8) | 26 (0.2) |  |
|  | Portacath | 74 (0.6) | 2 (0.4) | 72 (0.6) |  |
|  | Cordis/Introducer | 4110 (34.3) | 39 (7.5) | 4071 (35.5) |  |
|  | Multi Lumen | 7773 (64.8) | 474 (91.3) | 7299 (63.6) |  |
| Line Site, n (%) | Right Internal Jugular | 7967 (66.4) | 211 (40.7) | 7756 (67.6) | <0.001 |
|  | Right Femoral | 1151 (9.6) | 41 (7.9) | 1110 (9.7) |  |
|  | Left Internal Jugular | 1021 (8.5) | 117 (22.5) | 904 (7.9) |  |
|  | Left Subclavian | 743 (6.2) | 72 (13.9) | 671 (5.8) |  |
|  | Right Subclavian | 579 (4.8) | 63 (12.1) | 516 (4.5) |  |
|  | Left Femoral | 527 (4.4) | 15 (2.9) | 512 (4.5) |  |
|  | Central | 9 (0.1) | 0 (0.0) | 9 (0.1) |  |
|  | Right External Jugular | 1 (0.0) | 0 (0.0) | 1 (0.0) |  |
|  | Left External Jugular | 1 (0.0) | 0 (0.0) | 1 (0.0) |  |
| Gender, n (%) | Male | 4561 (38.0) | 215 (41.4) | 4346 (37.9) | 0.111 |
|  | Female | 7438 (62.0) | 304 (58.6) | 7134 (62.1) |  |
| Race, n (%) | White | 8070 (67.3) | 300 (57.8) | 7770 (67.7) | <0.001 |
|  | Black | 1028 (8.6) | 58 (11.2) | 970 (8.4) |  |
|  | Hispanic | 414 (3.5) | 31 (6.0) | 383 (3.3) |  |
|  | Asian | 312 (2.6) | 16 (3.1) | 296 (2.6) |  |
|  | others | 2175 (18.1) | 114 (22.0) | 2061 (18.0) |  |
| Marital status, n (%) | Married | 6189 (51.6) | 313 (60.3) | 5876 (51.2) | <0.001 |
|  | No married | 5810 (48.4) | 206 (39.7) | 5604 (48.8) |  |
| Insurance, n (%) | Medicare | 6622 (55.2) | 261 (50.3) | 6361 (55.4) | <0.001 |
|  | Private | 3322 (27.7) | 136 (26.2) | 3186 (27.8) |  |
|  | Medicaid | 1624 (13.5) | 90 (17.3) | 1534 (13.4) |  |
|  | others | 431 (3.6) | 32 (6.2) | 399 (3.5) |  |
| On ventilation, n (%) | No | 3030 (25.3) | 47 (9.1) | 2983 (26.0) | <0.001 |
|  | Yes | 8969 (74.7) | 472 (90.9) | 8497 (74.0) |  |
| Antibiotic prior 48h, n (%) | No | 4888 (40.7) | 87 (16.8) | 4801 (41.8) | <0.001 |
|  | Yes | 7111 (59.3) | 432 (83.2) | 6679 (58.2) |  |
| On vasopressor, n (%) | No | 5927 (49.4) | 169 (32.6) | 5758 (50.2) | <0.001 |
|  | Yes | 6072 (50.6) | 350 (67.4) | 5722 (49.8) |  |
| Arterial line, n (%) | No | 4266 (35.6) | 102 (19.7) | 4164 (36.3) | <0.001 |
|  | Yes | 7733 (64.4) | 417 (80.3) | 7316 (63.7) |  |
| On dialysis, n (%) | No | 10993 (91.6) | 338 (65.1) | 10655 (92.8) | <0.001 |
|  | Yes | 1006 (8.4) | 181 (34.9) | 825 (7.2) |  |
| Antibiotic types count, n (%) | 0 | 4888 (40.7) | 87 (16.8) | 4801 (41.8) | <0.001 |
|  | 1 | 3717 (31.0) | 68 (13.1) | 3649 (31.8) |  |
|  | 2 | 1528 (12.7) | 113 (21.8) | 1415 (12.3) |  |
|  | 3 | 1088 (9.1) | 109 (21.0) | 979 (8.5) |  |
|  | 4 | 489 (4.1) | 78 (15.0) | 411 (3.6) |  |
|  | 5 | 180 (1.5) | 33 (6.4) | 147 (1.3) |  |
|  | 6 | 77 (0.6) | 21 (4.0) | 56 (0.5) |  |
|  | 7 | 23 (0.2) | 5 (1.0) | 18 (0.2) |  |
|  | 8 | 7 (0.1) | 4 (0.8) | 3 (0.0) |  |
|  | 9 | 2 (0.0) | 1 (0.2) | 1 (0.0) |  |
| Concurrent lines, n (%) | 1 | 10873 (90.6) | 464 (89.4) | 10409 (90.7) | 0.014 |
|  | 2 | 1062 (8.9) | 47 (9.1) | 1015 (8.8) |  |
|  | 3 | 59 (0.5) | 8 (1.5) | 51 (0.4) |  |
|  | 4 | 4 (0.0) | 0 (0.0) | 4 (0.0) |  |
|  | 5 | 1 (0.0) | 0 (0.0) | 1 (0.0) |  |
| Catheter duration hours, median (IQR) |  | 48.00 (24.63-48.00) | 48.00 (48.00-48.00) | 46.65 (24.16-48.00) | <0.001 |
| Age(years), median (IQR) |  | 65.00 (55.00-75.00) | 61.00 (47.00-70.50) | 66.00 (55.00-75.00) | <0.001 |
| Hospital los days(days), median (IQR) |  | 10.30 (6.10-19.37) | 29.73 (19.23-45.98) | 9.95 (6.02-18.15) | <0.001 |
| ICU los days(days), median (IQR) |  | 3.52 (1.89-8.39) | 20.81 (13.28-31.96) | 3.32 (1.80-7.35) | <0.001 |
| Admission Weight(kg), median (IQR) |  | 80.90 (68.50-95.95) | 84.30 (70.00-101.30) | 80.70 (68.40-95.50) | <0.001 |
| BMI(kg/m²), median (IQR) |  | 27.97 (24.36-32.46) | 29.21 (24.91-34.42) | 27.92 (24.34-32.41) | <0.001 |
| Ventilation hours before line(hours), median (IQR) |  | 1.82 (0.00-49.36) | 273.60 (86.12-637.24) | 1.38 (0.00-42.22) | <0.001 |
| Temp avg(°F), median (IQR) |  | 98.37 (97.95-98.94) | 98.88 (98.24-99.57) | 98.35 (97.95-98.90) | <0.001 |
| Temp max(°F), median (IQR) |  | 99.20 (98.60-100.40) | 101.30 (99.80-102.50) | 99.20 (98.60-100.30) | <0.001 |
| Temp min(°F), median (IQR) |  | 97.60 (97.00-98.00) | 97.30 (96.00-97.80) | 97.60 (97.00-98.00) | <0.001 |
| Heart rate avg(bpm), median (IQR) |  | 83.85 (76.18-93.66) | 92.06 (83.09-101.02) | 83.53 (75.97-93.15) | <0.001 |
| Heart rate max(bpm), median (IQR) |  | 106.00 (93.00-121.00) | 126.00 (112.00-142.00) | 105.00 (92.00-120.00) | <0.001 |
| Sbp avg(mmHg), median (IQR) |  | 75.00 (70.54-80.74) | 75.21 (70.53-81.27) | 75.00 (70.54-80.70) | 0.459 |
| Dbp avg(mmHg), median (IQR) |  | 63.17 (56.31-72.10) | 65.09 (56.09-75.49) | 63.10 (56.32-71.91) | 0.006 |
| Map avg(mmHg), median (IQR) |  | 73.54 (67.55-80.28) | 73.24 (67.25-79.78) | 73.56 (67.57-80.29) | 0.326 |
| Resp rate avg(bpm), median (IQR) |  | 17.58 (15.59-20.03) | 19.97 (17.38-22.31) | 17.49 (15.54-19.90) | <0.001 |
| Spo2_avg(%), median (IQR) |  | 97.50 (96.38-98.49) | 97.49 (96.27-98.46) | 97.50 (96.39-98.50) | 0.304 |
| Spo2_min(%), median (IQR) |  | 92.00 (89.00-94.00) | 90.00 (84.00-92.00) | 92.00 (90.00-94.00) | <0.001 |
| Hematocrit(%), median (IQR) |  | 33.60 (30.10-37.60) | 33.30 (30.10-37.30) | 33.60 (30.10-37.70) | 0.479 |
| Hemoglobin(g/dL), median (IQR) |  | 11.10 (9.80-12.50) | 10.90 (9.80-12.35) | 11.10 (9.80-12.50) | 0.109 |
| Platelet(x10^9/L), median (IQR) |  | 14.30 (10.50-19.20) | 18.10 (12.95-24.50) | 14.10 (10.40-19.00) | <0.001 |
| Lactate(mmol/L), median (IQR) |  | 201.00 (149.00-277.00) | 273.00 (171.00-426.00) | 199.00 (149.00-272.00) | <0.001 |
| Creatinine(mg/dL), median (IQR) |  | 2.40 (1.70-3.50) | 2.90 (1.70-5.30) | 2.40 (1.70-3.40) | <0.001 |
| BUN(mg/dL), median (IQR) |  | 1.10 (0.80-1.70) | 1.70 (1.00-3.30) | 1.00 (0.80-1.60) | <0.001 |
| Sodium(mmol/L), median (IQR) |  | 22.00 (15.00-37.00) | 45.00 (26.00-75.00) | 21.00 (15.00-36.00) | <0.001 |
| Potassium(mmol/L), median (IQR) |  | 140.00 (138.00-143.00) | 143.00 (139.00-147.00) | 140.00 (138.00-143.00) | <0.001 |
| Calcium(mg/dL), median (IQR) |  | 4.40 (4.10-4.80) | 4.70 (4.30-5.20) | 4.40 (4.00-4.80) | <0.001 |
| PT(s), median (IQR) |  | 8.60 (8.10-9.10) | 8.80 (8.30-9.50) | 8.60 (8.10-9.10) | <0.001 |
| PTT(s), median (IQR) |  | 15.40 (13.70-17.80) | 16.90 (14.30-22.90) | 15.30 (13.70-17.70) | <0.001 |
| INR, median (IQR) |  | 35.70 (30.00-52.50) | 45.40 (31.68-74.83) | 35.40 (30.00-51.20) | <0.001 |
| Glucose(mg/dL), median (IQR) |  | 1.40 (1.20-1.60) | 1.50 (1.30-2.10) | 1.40 (1.20-1.60) | <0.001 |
| WBC(x10^9/L), median (IQR) |  | 148.00 (119.00-194.00) | 172.00 (141.00-234.00) | 146.00 (118.00-192.00) | <0.001 |
| Vasopressor dose(mcg/kg/min), median (IQR) |  | 14.30 (10.80-19.00) | 18.85 (13.80-25.80) | 14.10 (10.80-18.70) | <0.001 |
| Fluid input 24h before(mL), median (IQR) |  | 306606.47 (62990.57-1256387.57) | 5413799.13 (1854856.51-13514791.72) | 280428.37 (58115.65-1056350.05) | <0.001 |
| Fluid output 24h before(mL), median (IQR) |  | 115000.00 (12030.00-762140.00) | 3764763.00 (1105860.00-9466463.00) | 101150.00 (10430.00-624895.00) | <0.001 |
| Fluid input 24h after(mL), median (IQR) |  | 726258.58 (285445.95-2392967.98) | 9182189.28 (3592634.76-22077647.03) | 673185.32 (274162.71-2085367.21) | <0.001 |
| Fluid_output 24h after(mL), median (IQR) |  | 442722.00 (198447.50-1380994.50) | 5314800.00 (1926249.50-12633550.00) | 418750.00 (191377.50-1193983.75) | <0.001 |
| Daily charting frequency(times/day), median (IQR) |  | 70.47 (60.73-82.81) | 64.57 (58.50-73.37) | 70.75 (60.87-83.27) | <0.001 |
| Unique caregivers(n), median (IQR) |  | 11.00 (7.00-21.00) | 36.00 (26.00-49.00) | 10.00 (7.00-19.00) | <0.001 |
| **Abbreviations:** RIC, Rapid Infusion Catheter; PICC, Peripherally Inserted Central Catheter; Hickman, Hickman Catheter; Portacath, Implantable Port Catheter; Cordis/Introducer, Cordis or Introducer Sheath; Multi Lumen, Multi-lumen Central Venous Catheter; LOS, Length of Stay; BMI, Body Mass Index; SBP, Systolic Blood Pressure; DBP, Diastolic Blood Pressure; MAP, Mean Arterial Pressure; SpO₂, Peripheral Oxygen Saturation; WBC, White Blood Cell count; BUN, Blood Urea Nitrogen; PT, Prothrombin Time; PTT, Partial Thromboplastin Time; INR, International Normalized Ratio; Vasopressor dose, maximum dose of vasopressor (mcg/kg/min); Fluid input/output, total fluid input/output 24h before/after line insertion (mL); Concurrent lines, number of central lines present simultaneously; Ventilation hours before line, duration of mechanical ventilation prior to line insertion (hours); Daily charting frequency, number of nursing documentation events per day; Unique caregivers, number of distinct caregivers involved. | | | | | |

**Supplementary Table 2. Baseline characteristics of the patients.**

| **Models** | **AUC (95%CI)** | **Cutoff value** | **SEN (95%CI)** | **SPE (95%CI)** | **PLR (95%CI)** | **NLR (95%CI)** | **PPV (95%CI)** | **NPV (95%CI)** | **F1 score** | **Brier score** |
| --- | --- | --- | --- | --- | --- | --- | --- | --- | --- | --- |
| Logistic Regression | Logistic Regression | 0.908 (0.855–0.954) | 0.314 | 0.805 (0.681–0.914) | 0.852 (0.763–0.933) | 5.455 (3.376–12.213) | 0.229 (0.101–0.381) | 0.786 (0.666–0.894) | 0.867 (0.768–0.936) | 0.795 |
| Decision Tree | Decision Tree | 0.820 (0.744–0.897) | 1.000 | 0.902 (0.800–0.977) | 0.738 (0.619–0.855) | 3.441 (2.340–6.291) | 0.132 (0.032–0.272) | 0.698 (0.558–0.820) | 0.918 (0.833–0.982) | 0.787 |
| Random Forest | Random Forest | 0.941 (0.893–0.982) | 0.510 | 0.927 (0.844–1.000) | 0.836 (0.742–0.931) | 5.654 (3.565–12.891) | 0.088 (0.000–0.197) | 0.792 (0.674–0.911) | 0.944 (0.879–1.000) | 0.854 |
| XGBoost | XGBoost | 0.918 (0.860–0.958) | 0.667 | 0.951 (0.872–1.000) | 0.836 (0.738–0.919) | 5.802 (3.558–11.912) | 0.058 (0.000–0.149) | 0.796 (0.673–0.889) | 0.962 (0.898–1.000) | 0.867 |
| SVM | SVM | 0.919 (0.855–0.966) | 0.334 | 0.902 (0.805–0.979) | 0.787 (0.704–0.873) | 4.235 (2.925–7.204) | 0.124 (0.027–0.256) | 0.740 (0.613–0.851) | 0.923 (0.842–0.981) | 0.813 |
| Artificial Neural Network | Artificial Neural Network | 0.946 (0.899–0.981) | 0.434 | 0.951 (0.868–1.000) | 0.869 (0.786–0.947) | 7.253 (4.452–18.591) | 0.056 (0.000–0.148) | 0.830 (0.717–0.925) | 0.964 (0.907–1.000) | 0.886 |
| Gradient Boosting | Gradient Boosting | 0.910 (0.852–0.961) | 0.255 | 0.902 (0.791–0.979) | 0.787 (0.679–0.882) | 4.235 (2.763–7.506) | 0.124 (0.025–0.254) | 0.740 (0.628–0.840) | 0.923 (0.833–0.984) | 0.813 |

**Supplementary Table 3. Supplementary Table 3. Sensitivity analysis excluding patients with arterial catheterization.**

| **Feature** | **Coefficient** | **Abs_Coefficient** |
| --- | --- | --- |
| icu_los_days | 0.049756315 | 0.049756315 |
| ventilation_hours_before_line | 0.022255123 | 0.022255123 |
| on_dialysis | 0.010745275 | 0.010745275 |
| fluid_output_24h_before | 0.00923085 | 0.00923085 |
| antibiotic_types_count | 0.007917643 | 0.007917643 |
| bun | 0.007798942 | 0.007798942 |
| heart_rate_avg | 0.005481433 | 0.005481433 |
| lactate | 0.003584997 | 0.003584997 |
| wbc_48h | 0.002370286 | 0.002370286 |
| age | -0.000772371 | 0.000772371 |
| temp_max | -0.000597898 | 0.000597898 |
| resp_rate_avg | 0.000392041 | 0.000392041 |
| arterial_line | 0.000254779 | 0.000254779 |
| unique_caregivers | 4.50169E-05 | 4.50169E-05 |
| fluid_output_24h_after | 3.81531E-05 | 3.81531E-05 |
| line_type | 0 | 0 |
| hospital_los_days | 0 | 0 |
| catheter_duration_hours | 0 | 0 |
| concurrent_lines | 0 | 0 |
| marital_status | 0 | 0 |
| insurance | 0 | 0 |
| on_ventilation | 0 | 0 |
| antibiotic_prior_48h | 0 | 0 |
| on_vasopressor | 0 | 0 |
| line_site | 0 | 0 |
| race | 0 | 0 |
| bmi | 0 | 0 |
| dbp_avg | 0 | 0 |
| heart_rate_max | 0 | 0 |
| temp_min | 0 | 0 |
| temp_avg | 0 | 0 |
| admission_weight | 0 | 0 |
| creatinine | 0 | 0 |
| platelet | 0 | 0 |
| spo2_min | 0 | 0 |
| wbc | 0 | 0 |
| pt | 0 | 0 |
| sodium | 0 | 0 |
| calcium | 0 | 0 |
| potassium | 0 | 0 |
| glucose | 0 | 0 |
| inr | 0 | 0 |
| ptt | 0 | 0 |
| wbc_baseline | 0 | 0 |
| fluid_input_24h_before | 0 | 0 |
| vasopressor_dose | 0 | 0 |

**Supplementary Table 4. LASSO coefficients all.**
